# Thyroid ultrasound findings in a follow-up survey of children from three Japanese prefectures: Aomori, Yamanashi, and Nagasaki

**DOI:** 10.1038/srep09046

**Published:** 2015-03-12

**Authors:** Naomi Hayashida, Misa Imaizumi, Hiroki Shimura, Fumihiko Furuya, Noriyuki Okubo, Yasushi Asari, Takeshi Nigawara, Sanae Midorikawa, Kazuhiko Kotani, Shigeyuki Nakaji, Akira Ohtsuru, Takashi Akamizu, Masafumi Kitaoka, Shinichi Suzuki, Nobuyuki Taniguchi, Shunichi Yamashita, Noboru Takamura

**Affiliations:** 1 Division of Strategic Collaborative Research, Center for promotion of collaborative research on radiation and environment health effects, Atomic Bomb Disease Institute, Nagasaki University, Nagasaki, Japan; 2 Department of Global Health, Medicine and Welfare, Atomic Bomb Disease Institute, Nagasaki University, Nagasaki, Japan; 3 Department of Radiation Medical Sciences, Atomic Bomb Disease Institute, Nagasaki University, Nagasaki, Japan; 4 Department of Clinical Studies, Radiation Effects Research Foundation, Nagasaki, Japan; 5 The Third Department of Internal Medicine, Faculty of Medicine, The University of Yamanashi, Yamanashi, Japan; 6 Department of Social Medicine, Hirosaki University Graduate School of Medicine, Hirosaki, Aomori, Japan; 7 Department of Emergency and Disaster Medicine, Hirosaki University Graduate School of Medicine, Hirosaki, Aomori, Japan; 8 Department of Endocrinology and Metabolism, Hirosaki University Graduate School of Medicine, Hirosaki, Aomori, Japan; 9 Department of Laboratory Medicine, Fukushima Medical University, Fukushima, Japan; 10 Department of Radiation Health Management, Fukushima Medical University, Fukushima, Japan; 11 Department of Thyroid and Endocrinology, Fukushima Medical University, Fukushima, Japan; 12 Radiation Medical Science Center for the Fukushima Health Management Survey, Fukushima Medical University, Fukushima, Japan; 13 Department of Clinical Laboratory Medicine, Jichi Medical University, Tochigi, Japan; 14 The First Department of Medicine, Wakayama Medical University, Wakayama, Japan; 15 Division of Endocrinology and Metabolism, Showa General Hospital, Tokyo, Japan

## Abstract

We conducted ultrasound thyroid screening in cohort of 4,365 children aged between 3 to 18 years in three Japanese prefectures (Aomori, Yamanashi, and Nagasaki) using the same procedures as used in the Fukushima Health Survey. Forty-four children had nodules ≥ 5.1 mm in diameter or cysts ≥ 20.1 mm in diameter detected at the first screening, and 31 of these children underwent the second follow-up survey. We collected information from thyroid ultrasound examinations and final clinical diagnoses and re-categorized the thyroid findings after the second examination. Twenty children had nodules ≥ 5.1 mm in diameter or cysts ≥ 20.1 mm in diameter at the second examination; of these, one child was diagnosed with a thyroid papillary carcinoma and the remaining 19 children were diagnosed with possibly benign nodules such as adenomas, adenomatous nodules, and adenomatous goiters. A further 11 children were re-categorized as “no further examinations were required.” Our results suggest that ultrasound thyroid findings in children may change with a relatively short-term passing period, and that thyroid cancer may exist at a very low but certain frequency in the general childhood population.

The accident at the Fukushima Dai-ichi Nuclear Power Plant (FNPP), which occurred due to the Great East Japan Earthquake on March 11, 2011, released a huge amount of radioactive material into the environment and generated a wide, diffuse radioactive plume. Residents of Fukushima Prefecture therefore experienced disaster damage from three sources: the earthquake, the tsunami, and the nuclear accident.

After the accident, residents living in a 30 km range around the FNPP were evacuated. Three years have passed since the accident, but many residents of Fukushima remain evacuated both inside and outside the Fukushima prefecture in Japan. Since the initial phase of the accident, the Japanese Government has conducted, in addition to the official order of prompt evacuation, “food control,” in the form of prohibition of distribution and consumption of contaminated foods and water, to minimize radiation exposure, especially internal exposure to the thyroid gland due to radioiodine such as iodine-131 (^131^I)^1^.

Data are limited regarding the measurements of radiation exposure to the thyroid in children at the initial phase of the accident. During March 26 to 30, 2011, two weeks after the accident, thyroid monitoring using a NaI (TI) scintillation survey meter was conducted on children ranging from 0 to 15 years of age: 315 were from Iitate Village, 631 from Kawamata Town, and 137 from Iwaki City. The survey showed that 95.7% of the children had received < 10 mSv, with a maximum of 35 mSv, which was lower than the intervention level (50 mSv)^2^,^3^. Tokonami et al. also measured ^131^I-activity in the thyroid glands of 62 residents and evacuees during the period from April 12 to 16, 2011, and found that thyroid equivalent doses by inhalation ranged from none detected to 33 mSv, with a median thyroid equivalent dose for children and adults of 4.2 and 3.5 mSv, respectively^4^. The regions where these direct measurements were carried out are considered to be areas of high risk for thyroid internal exposure following the FNPP accident. These results suggest that the countermeasures taken at the outset of the accident in Fukushima effectively minimized the internal radiation exposure of the thyroid gland.

Nevertheless, possible adverse health effects to the thyroid glands of children due to radiation exposure have been a concern in Fukushima. The Fukushima Prefecture launched the Fukushima Health Survey, which targeted all residents living in that Prefecture^5^. The purpose of this survey was to monitor the long-term health of residents, promote their future well-being, and clarify whether long-term low-dose radiation exposure had adverse health effects. The survey consisted of a basic survey and four detailed surveys that included a thyroid ultrasound examination, a comprehensive health check, a mental health and lifestyle survey, and a pregnancy and birth survey^6^. The thyroid ultrasound examination targeted all prefectural inhabitants aged between 0 to 18 years at the time of the FNPP accident and identified small nodules (≤5.0 mm) or cysts (≤20.0 mm) in about 45% (136,804/295,511) of the target inhabitants^7^. A further 90 cases showed malignancy or suspected malignancy by cytology or surgery. Of these 90 cases, although the ratio according to the size of nodules is not reported, the average size is relatively small, at 14.2 ± 7.4 mm^7^. The radiation exposure dose to the thyroid glands of Fukushima residents was estimated to be limited; however, the possibility should be considered that the observed thyroid disorders were induced even by this low dose radiation exposure.

Recently, we investigated the frequency of thyroid nodules or cysts in the general population of Japanese children by conducting ultrasound thyroid screening using the same procedures as was used in the Fukushima Survey. We examined 4,365 children aged between 3 and 18 years in three Japanese prefectures (Aomori, Yamanashi and Nagasaki) between November 2012 and January 2013^8^,^9^. In these studies, of the 4,365 children, 1,853 participants (42.5%) had no nodules or cysts. We detected thyroid nodules ≤ 5.0 mm in diameter or cysts ≤ 20.0 mm in diameter in 2,468 participants (56.5%) and nodules ≥ 5.1 mm in diameter or cysts ≥ 20.1 mm in diameter in 44 participants (1.0%). We further reported the frequency of ultrasonographic thyroid findings such as nodules, cysts, diffuse goiter, ectopic thymus, or ultimobranchial body.

Clarification of the clinical diagnosis of these nodules required further examination of the children in these prefectures. Therefore, the aim of the present study was to investigate the clinical diagnosis of thyroid nodules detected in the first screening and to summarize the results of further thyroid examinations by conducting a follow-up survey of children who underwent a second thyroid examination.

## Results

The age of the 31 children at the second examination was 4–19 years old (mean ± standard deviation (SD), 14.5 ± 3.1 years old) and most children (28/31) were ≥10 years old. Seven children were males and 24 were females. The second thyroid examinations were conducted between March 2013 and March 2014; the intervals between the first examinations and the second examinations were 2–15 months (5 ± 3 months).

Thyroid cysts and nodules were detected in 19 and 22 participants, respectively. Fourteen participants had both nodules and cysts. The maximum diameter ranged from 2 mm to 11 mm (6 ± 3 mm) for the cysts and from 5 mm to 24 mm (9 ± 4 mm) for the nodules. Diffuse goiter was identified in 3 children and cervical lymph node swelling was identified in 1 child.

**Figure 1:**
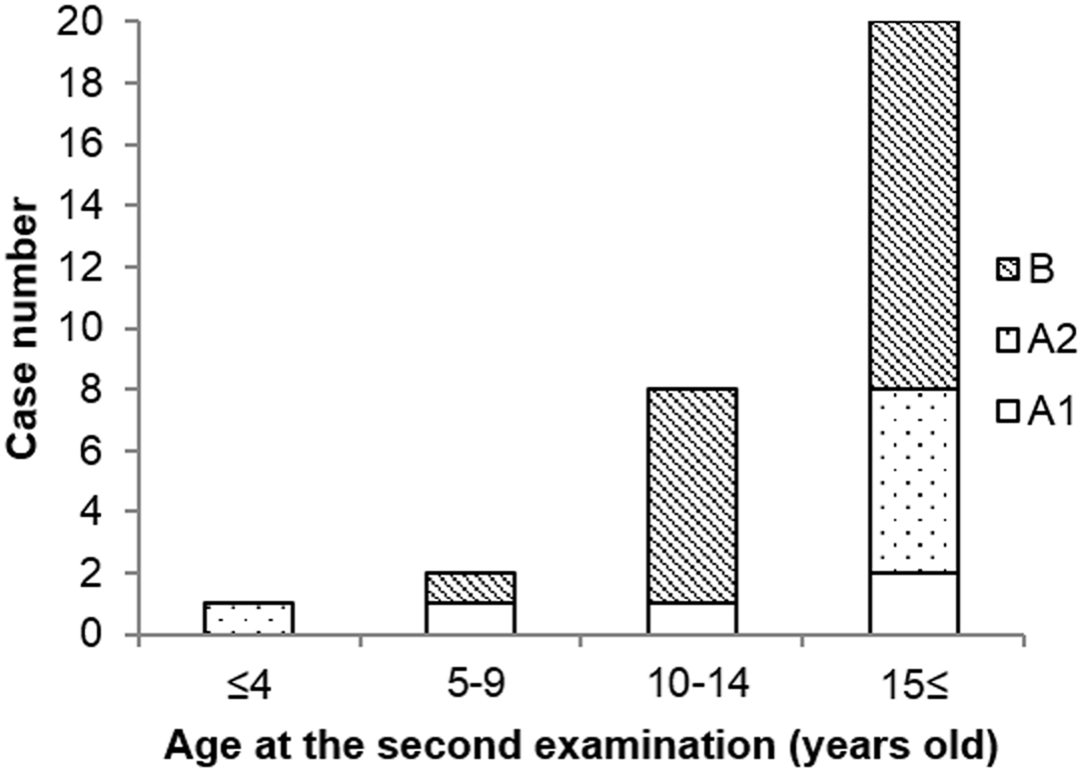
Case numbers of categorized thyroid findings classified by age of participants. Categories: ‘A1’, no nodules or cysts; ‘A2’, the presence of nodules ≤ 5.0 mm in diameter or cysts ≤ 20.0 mm in diameter; ‘B’, the presence of nodules ≥ 5.1 mm in diameter or cysts ≥ 20.1 mm in diameter.

Table 1 shows the number of participants classified according to their categorized thyroid findings. Overall, one third of children were classified with a status of ‘A’ in the second examination. No children were classified with a status of ‘C’. Figure 1 shows the case numbers of categorized thyroid findings classified by the age at the second examination. Most children classified with a status of ‘B’ (19/20) were ≥10 years old.

Among 4 children classified with a status of ‘A1’, 3 children had normal thyroid findings and 1 participant had diffuse goiter. In 2 of the 3 children with normal thyroid findings, any nodules and/or cysts detected at the first examinations were confirmed as artifacts from vessels. In the third participant, a cyst identified at the first examination had disappeared by the second examination. One child who had multiple hypoechoic lesions in the thyroid at the first examination was identified as having the heterogenous diffuse goiter of Graves' disease because thyroid function was high and blood tests revealed positive thyroid stimulating antibodies (TSAb).

Among 7 children classified with a status of ‘A2’, 2 children had both a nodule with a maximum diameter of ≤5 mm and a cyst with a maximum diameter of ≤20 mm, while 5 participants had cysts with a maximum diameter of ≤20 mm.

All 20 children classified with a status of ‘B’ had nodules and 12 children also had cysts. Aspiration biopsies and cytological examinations were performed for 2 participants based on decisions of thyroid expert medical doctors. These doctors suspected malignancy because internal echoes of these nodules were heterogeneous. No malignant cells were detected in the nodule of 1 of these 2 children. An inadequate sample was obtained from the nodule of the other child (a female ≥ 15 years old). The ultrasonographic features of her nodule were suspicious for malignancy and she had swelling of a cervical lymph node of the lateral neck, so she underwent thyroid surgery and was diagnosed with papillary carcinoma.

**Figure 2:**
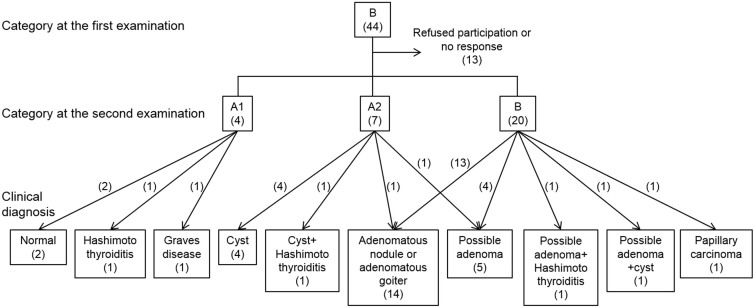
Breakdown of participants into their clinical diagnoses. The numbers in parentheses indicate the numbers of participants. Categories: ‘A1’, no nodules or cysts; ‘A2’, the presence of nodules ≤ 5.0 mm in diameter or cysts ≤ 20.0 mm in diameter; ‘B’, the presence of nodules ≥ 5.1 mm in diameter or cysts ≥ 20.1 mm in diameter. The ultrasonographic diagnosis was determined by ultrasonographic findings and the clinical diagnosis was determined by not only ultrasonographic findings but also other examinations such as blood tests and cytological examinations.

Figure 2 shows the breakdown of the children into their clinical diagnoses. Among the 20 children whose nodules were categorized as ‘B’, 19 were diagnosed with possibly benign nodules such as adenomas, adenomatous nodules, or adenomatous goiters based on the ultrasonographic features of their nodules. (The remaining child was the one described above who was diagnosed with papillary carcinoma.) Among 7 children with cysts or nodules categorized as ‘A2’, 5 were diagnosed with cysts and 2 were diagnosed with possibly benign nodules. One child was diagnosed as having Graves' disease based on a positive result for thyroid stimulating antibody and 3 children were diagnosed as having Hashimoto's thyroiditis based on positive results of anti-thyroglobulin antibody and anti-thyroid peroxidase antibody. Clinical follow-ups of 2 children with normal thyroids and one participant whose cyst decreased to 2 mm in diameter were discontinued after the second examinations.

## Discussion

In this study, we investigated the detailed results of further thyroid examinations performed in hospitals (second thyroid examinations) on children classified with a status of ‘B’. A total of 31 children underwent a second thyroid examination; 11 children were classified with a status of ‘A’ in the second examination, while no child was classified with a status of ‘C’. The changes in classification for thyroid findings in the 11 children occurred 2–15 months after the initial examinations.

One of the reasons for these types of changes may be differences in the imaging quality of the ultrasound examinations. The first thyroid screenings were conducted using 7.75-MHz ultrasound probes (12L-RS linear array transducer [GE Health care, Japan] and LOGIQ e Expert ultrasound [GE Healthcare, Japan]), and the machine and settings were the same as those used in the Fukushima Health Survey. Furthermore, if a finding with a diameter ≥ 5.1 mm was suspected as cyst but its contents could not be sufficiently evaluated, it was regarded as a nodule and classified with a status of ‘B’. On the other hand, as in the Fukushima Health Survey, children underwent thyroid ultrasonography using the various types of devices available at each hospital, but the ultrasound imaging quality was higher for the second thyroid examination than for the first screening where a portable ultrasound was used. Two of the 4 children who were classified with a status of ‘A1’ at the second screening had their nodules and/or cysts detected at the initial examinations confirmed as artifacts from vessels. Another of these 4 children had multiple hypoechoic lesions in the thyroid at the initial examination and was subsequently identified as having heterogenous diffuse goiter of Graves' disease. Five of the 7 children classified with a status of ‘A2’ at the second screening had only cysts with a maximum diameter of ≤20 mm without nodules.

Another possible reason is that the observed changes are typical thyroid findings in children. A few reports have been published regarding the natural history of benign thyroid nodules based on ultrasonographic evaluation. For example, Erdogan et al. collected retrospective data for 531 nodules in 420 patients who were followed by ultrasonography for a mean duration of 39.7 ± 27.8 months (range 12-168 months). These researchers reported that one third of the 531 nodules showed continuous growth, one third remained unchanged, and the other third decreased in size^10^. Lim et al. also described the natural course of benign thyroid nodules in 186 patients, and showed that nodule volumes increased in 11.8%, underwent no change in 79.9%, and decreased in 8.3% after a mean duration of 21.7 ± 10.7 months^11^. On the other hand, the long-term natural history of benign thyroid nodules in children has not been extensively studied. Previously, we reported the prognosis of thyroid nodules in individuals living in the Zhitomir Region of Ukraine aged 0 to 10 years old at the time of the accident, and showed that nodule number and size were significantly increased in the second compared with the first screening (median follow-up period 172 months)^12^. In our study, the maximum diameter of the nodules was ≥5.1 mm in the first examinations, but 2 children had a nodule with a maximum diameter of ≤5 mm and were classified with a status of ‘A2’ in the second examination.

Two of the 20 children who had nodules classified as a status of ‘B’ underwent aspiration biopsies and cytological examinations, but an inadequate sample was obtained from the nodule of one of these participants. Based on the ultrasonographic features of the nodule, she underwent thyroid surgery and was diagnosed with papillary carcinoma based on histological evaluation. Aghini-Lombardi et al. reported that thyroid nodularity was 0.5% in children (<15 years old) and progressively increased with age to 2.1% in the group aged 15 to 25 years^13^. The incidence of differentiated thyroid carcinoma in children was reported by Vergamini et al. as an age-standardized rate according to age groups of 0–4, 5–9, 10–14, and 15–19 years of age with values of 0.04, 0.43, 3.50, and 15.16 per million, respectively^14^. Furthermore, the incidence of thyroid carcinoma in 2006 in the Russian Federation was reported to vary considerably with age and gender, according to the official data. In the Russian report, the crude incidence was 0.01 per 100,000 population in the 0–4 year age group, 0.05 in the 5–9 year age group, 0.37 in 10–14 year age group, and 0.86 in 15–19 year age group, and 1.86 in males and 10.07 in females^15^. In the present study, we identified one case of papillary carcinoma in a female who was more than 15 years old. The recent Fukushima Health Survey report provided results for 295,511 children aged 0 to 18 years old at the time of the accident who underwent thyroid ultrasound examination. Of these, 49 children were diagnosed with papillary carcinoma based on histological evaluation^7^. Our present results on the frequency of thyroid carcinoma are similar to the findings of this survey report^7^. However, the numerical values cannot be directly compared between the two surveys without careful consideration of the different backgrounds of the study populations (e.g., different iodine intake levels). In our study, sample size was limited, but our results suggest that childhood thyroid cancer may exist at a certain frequency in the general population. Few studies have focused on generic thyroid ultrasound screenings in children; therefore, the results presented here for the frequency of papillary thyroid carcinoma in this generation are informative.

Our study has several limitations. First, not of all children identified with a status of ‘B’ agreed to participate in this research. We invited 44 children, but only 31 children participated in the study, which might have caused sampling bias. Furthermore, the indications for performing aspiration biopsies and cytological examinations were decided by each thyroid expert medical doctor, and these examinations were performed for only 2 children. Thus, most nodules were diagnosed as possibly benign nodules such as adenomas, adenomatous nodules, and adenomatous goiters based on the ultrasonographic feature of nodules, and not by histopathological evaluation.

In conclusion, we conducted a follow-up survey of a previously studied population who had been classified with a status of ‘B’; one third of these participants were classified with a status of ‘A’ in the second examination. These results indicate that ultrasound thyroid findings in children may change with a relatively short-term passing period, and that thyroid cancer may exist at a very low but certain frequency in the general childhood population. Careful thyroid ultrasound examinations would provide valuable data for understanding the thyroid findings in children, but thyroid ultrasound screening detects extremely small nodules or thyroid cysts that need not be treated clinically. Therefore, we advise that sufficient care be taken when conducting thyroid ultrasound screening, especially in children in the general population. The planning of thyroid ultrasound screening for children should also take into consideration the additional uneasiness that this might cause for the children and their families due to findings that need not be treated clinically.

## Methods

### Ethics Statement

This study was approved by the ethics committees of Hirosaki University, Yamanashi University, and Nagasaki University Hospital. It was conducted in accordance with the guidelines expressed in the Declaration of Helsinki. Written informed consent was obtained from the parents of all subjects.

### Study Area

This follow-up survey was conducted in the same areas as the first ultrasound examination; i.e., in Aomori Prefecture, Yamanashi Prefecture, and Nagasaki Prefecture, Japan. These areas were chosen because they have thyroid ultrasound specialists and medical facilities that enabled further examination. These areas are also geographically dispersed throughout the eastern, central, and western regions of Japan, and are thought to have been unaffected by radioactive material from the FNPP accident^8^,^9^.

### Study Population

The first ultrasound thyroid screenings were performed between November 2012 and January 2013 in Japanese children aged 3 to 18 years, from one kindergarten, one elementary school, one junior high school, and one high school in each prefecture. The first screening investigated 4,365 children (Aomori: 1,630 children; Yamanashi: 1,366 children; Nagasaki: 1,369 children) using the same procedures as used in the Fukushima Health Survey. Of these, 44 children were classified with a status of ‘B’ (indicating the presence of nodules ≥ 5.1 mm in diameter or cysts ≥ 20.1 mm in diameter) and were recommended for further examinations (the second thyroid examinations)^8^.

In this study, these 44 children were invited to participate in the follow-up survey. We sent a set of documents to request participation in this research to the parents of the 44 children, and asked them to return signed documents when they agreed to participate in the research. The parents of 13 children refused to participate in the survey or did not respond to our invitation, so 31 children ultimately participated in this follow-up survey.

### Collection of clinical information

All participants in this study were recommended to undergo further thyroid examinations in hospital after the first examination. They were able to personally select the hospital at which they underwent further thyroid examination (the second thyroid examinations).

In the present study, we collected detailed information regarding the results of these second thyroid examinations for 31 children. We requested this information directly from the medical doctors who conducted the second thyroid examinations, and collected information including the age at the second examination, the date of the first and the second examinations, findings of the second thyroid ultrasound examinations, the categorization of the thyroid findings at the second examination, the clinical diagnoses, and the treatment procedures or the clinical management plans. We also collected results for children who underwent aspiration biopsies or cytological examinations, which were conducted when a malignancy was suspected or when the attending physician judged it to be necessary. The findings of the second thyroid ultrasound examinations included the presence of thyroid nodules or cysts, the maximum diameter of the thyroid nodules and cysts, and other thyroid findings (e.g., diffuse goiter, congenital thyroid defect, parathyroid swelling, lymph node swelling, ectopic thymus, or ultimobranchial body).

### Classification of thyroid findings

In this study, we used the same categories as were used in previous studies to classify the findings of thyroid nodules and cysts: category ‘A’ (‘A1’ and ‘A2’), ‘B’ or ‘C’. ‘A1’ was defined as the absence of nodules or cysts, ‘A2’ was defined as the presence of nodules ≤ 5.0 mm in diameter or cysts ≤ 20.0 mm in diameter, ‘B’ was defined as the presence of nodules ≥ 5.1 mm in diameter or cysts ≥ 20.1 mm in diameter, and ‘C’ was defined as the presence of thyroid findings that required immediate further examinations^6^,^8^,^9^.

## Figures and Tables

**Table 1:** Number of participants classified according to the categorized thyroid findings

Categories	Number
A	11 (35.5%)
A1	4 (12.9%)
A2	7 (22.6%)
B	20 (64.5%)
C	0
Total	31

The numbers in parentheses indicate the percentage of the total participants. Categories: ‘A’, no further thyroid examinations were required; ‘B’, the presence of nodules ≥ 5.1 mm in diameter or cysts ≥ 20.1 mm in diameter; ‘C’, the presence of thyroid findings requiring immediate further examinations in a hospital. Sub-categories: ‘A1’, no nodules or cysts; ‘A2’, the presence of nodules ≤ 5.0 mm in diameter or cysts ≤ 20.0 mm in diameter.
